# Regulations to reduce risk of hearing damage in concert venues

**DOI:** 10.2471/BLT.19.242404

**Published:** 2020-04-07

**Authors:** Elizabeth Francis Beach, Robert Cowan, Johannes Mulder, Ian O’Brien

**Affiliations:** a National Acoustic Laboratories, Australian Hearing Hub, 16 University Avenue, Macquarie University NSW 2109, Sydney, Australia.; b Department of Audiology and Speech Pathology, University of Melbourne, Melbourne, Australia.; c School of Arts, Murdoch University, Perth, Australia.; d Queensland Conservatorium of Music, Griffith University, Brisbane, Australia.

In 2015, the World Health Organization (WHO) convened a group of researchers and industry experts to tackle the risk of noise-induced hearing loss from loud music exposure because of personal listening devices and entertainment venues. The *Make Listening Safe* initiative has recently turned its attention to regulatory processes aimed at the protection of hearing at music venues. WHO estimates that 1.1 billion young people are at risk from recreational sound exposure, with attendees at concerts, nightclubs and bars exposed to sound levels that regularly exceed a continuous equivalent sound level of 100 dB.^1^ This degree of exposure puts regular attendees at risk of developing disabling hearing loss and permanent tinnitus. These conditions adversely affect an individual’s ability to communicate, leading to social isolation and poor educational and employment outcomes. Such outcomes have a significant impact at the individual and societal level.^2^

Although some European jurisdictions have introduced regulations, laws or standards to address the risk of hearing damage from music venues, most countries have not done so.^3^ In this paper, we outline several issues that need to be considered when considering implementation of a regulatory framework for music venues. Obviously, new regulations will need to respect existing regulations that also deal with sound levels and licensing of venues. Enforcement of regulations and the cost of compliance are key considerations that will impact on the implementation of regulations by authorities and venue managers. Finally, education, training and awareness-raising will likely be integral to ensuring the successful implementation of regulations.

It is important to consider how new regulations for protection of patrons’ hearing at entertainment venues fit within the existing regulatory framework within which venues operate. For example, in most jurisdictions, there are workplace health and safety laws that govern sound exposure for workers and there are upper sound-level limits set down by environmental law. In addition, venues are often subject to licensing arrangements, which, together with environmental sound regulations, are usually the responsibility of a municipal authority or local council. In contrast, occupational health and safety laws are more often within the function of state or federal governments.

The involvement of multiple levels of government, and different rules and regulations, increases the complexity associated with achieving compliance and can lead to considerable confusion for music venues. Successfully navigating the regulatory infrastructure to ensure that any new regulations are consistent with pre-existing rules will involve cooperation between various groups. It is notable that the European countries with some of the most highly developed systems for managing entertainment sound levels are the result of joint action by policy-makers and music industry representatives. In the Netherlands for example, a covenant has been in place since 2014, and although it has no legal status, it is the result of a collaboration between the Dutch Ministry for Health, Welfare and Sport and the two main national music industry bodies (the Association of Event Makers and the Association of Dutch Pop Music Venues and Festivals).^4^ Other countries, such as Norway and Austria, have taken a combined approach to the control of sound at entertainment venues by publishing both environmental and hearing health guidelines in a single document. The aim is to help venue operators understand what they need to do to comply with the multiple regulations which apply.^5^^,^^6^

It is important, however, to consider the issue of enforcement when devising regulations for entertainment venues, and whether regulations can be effective without accompanying enforcement by a local authority. In San Francisco, for example, where earplugs must be available in music venues of a certain size, there is no enforcement by local police, and yet compliance with regulations by 13 out of 18 venues was reported in 2015.^7^ This is likely due to the fact that the requirement to supply earplugs has been in place since 2002, it is a relatively low-cost measure and is easy to implement. In Germany, where more extensive, but voluntary, sound level guidelines are in place, researchers found that sound levels in 16 out of 20 indoor dance-music venues exceeded the sound level limit.^8^ When sound levels in entertainment venues are enforced, compliance rates seem to be better. For example, a study of dance music festivals in Belgium, found high compliance rates^9^ and a Swiss study found that 258 (87%) of 296 measurements taken at concerts held during a music festival were compliant.^10^ Both studies were conducted at outdoor music festivals, where there is a long tradition of monitoring and enforcing (environmental) sound levels, which may go some way towards explaining the higher rates of compliance at these types of events.

Enforcement brings with it several challenges, many of which are dependent on the requirements of the regulation. In Belgium, for example, sound level measurements must comply with detailed guidelines^11^ that require enforcement officers to record measurements over two periods (15- and 60-minute measurements are specified). The measurements must be uninterrupted, in a position that is representative of the audience exposure, and made using a class I sound level metre (International Electrotechnical Commission standard 61672) that is calibrated immediately before and after the procedure. Officers must operate covertly to avoid venue staff lowering the sound level when they notice the officers. Furthermore, the measurements should not include intentional heckling and excessive crowd noise (which, with a sizeable crowd, can easily exceed expected sound levels). Both these factors can contaminate a measurement and render it invalid. Consequently, in addition to the sound level data, an audio recording may also be required to ascertain whether the sound level was reduced by the operator when compliance officers were present or whether a sound level breach was an inadvertent result of audience noise (rather than the music).

The alternative to enforcement is implementation of voluntary guidelines with little or no external oversight or audit. In this case, it is likely that some venues will simply ignore the guidelines, since there is no penalty for failing to comply. In other cases, venues that are already subject to enforceable environmental sound limits (in which compliance is a pre-condition for a venue permit) will continue to focus on meeting the pre-existing limits.

To comply with regulations, venues will likely incur some costs. For example, venues may be required to purchase, install and regularly calibrate sound level meters, other sound system enhancements or other monitoring equipment. Venues may need to engage acoustic consultants to advise on measurement procedures (and in some cases may be required to do so before applying for a permit). Venue operators also need to supply earplugs, and in some cases, they may be required to reconfigure their venues to provide quiet spaces or restrict access to speakers, which could be expensive. A programme of financial incentives and a staged introduction of the new regulations would allow venues the necessary time and resources to comply with the new regulations.

For enforcement agencies, there are also costs for staffing, training and acquisition and calibration of equipment. Taking measurements, storing data, issuing warnings, penalty notices and fines all incur costs and consume time for the enforcing authority. These issues will need to be considered when deciding whether and how to enforce new hearing health- related regulations in entertainment venues.

Ideally, the introduction of new regulations would be supported with appropriate training for venue staff involved in managing sound levels and implementing the regulations, that is sound engineers and venue managers. In Germany, for example, a 3-hour tutorial was offered to disc jockeys when voluntary sound level limits were introduced. The training included instruction on the physics of sound, the physiology of the ear and the impact of loud music on hearing, and technical issues related to sound level monitoring and measurement.^8^ Although the training did not necessarily lead to reduced sound levels in venues, there is a strong case for providing sound engineers with the necessary knowledge and skills to manage sound levels and keep within the specified limits. Musicians, venue managers and others responsible for the sound levels would also benefit from training and information about sound levels in general and the impact of new regulations. For sound engineers, the training should be practical and hands-on, with a strong focus on techniques for reducing sound levels while maintaining the perception of loudness, such as increasing the volume of the bass and adding vibro-tactile feedback.^12^ Care is needed however, to ensure that these techniques do not encroach on co-existing environmental sound limits. In an ideal world, training and accreditation of sound engineers, along with regular check-ups on their hearing health, would be included as part of a regulation, perhaps as a pre-condition for obtaining a venue permit.

To support the introduction of regulations in venues, it will also be important to educate patrons about the risks associated with loud music exposure. There are several non-profit education campaigns, which, when combined with advocacy from high-profile musicians (often with acquired music-induced hearing loss themselves), can be a powerful motivational tool. Australia’s *Hearsmart*,^13^ the *I Love My Ears* campaign in the Netherlands^14^ and the United States-based *Hearing Education and Awareness for Rockers*^15^ are examples of initiatives that encourage individuals to prioritize their hearing health. Ideally, regulations and awareness-raising campaigns like these will work together to promote adoption of hearing health measures in venues by all stakeholders: managers, sound engineers, musicians and patrons.

Regulations for entertainment venues are in place in some countries and there are moves to consider introducing similar regulations in other markets. We believe that regulations should be considered as part of a wider solution that also includes education and awareness-raising for patrons, musicians, sound engineers and venue staff. Regulations should be simple, practical, easily understood and well-integrated with existing regulatory frameworks for environmental and occupational sound exposure. Enforcement procedures and cost implications will also need to be addressed. Ideally, the development of new regulations will have the support and involvement of multiple stakeholders who believe in the importance of reducing hearing risks in entertainment venues. All those involved will need to be committed to supporting the implementation process, despite the widespread attitude in the music industry that louder sound is better. The stakeholders will need to work together to identify appropriate sound level limits and practical hearing health measures that are achievable across a wide range of venue types, from large multiday music festivals to small inner-city music venues.
