# Corrigendum

**DOI:** 10.2471/BLT.20.100920

**Published:** 2020-09-01

In: Beach Elizabeth Francis, Cowan Robert, O’Brien Ian. Regulations to reduce risk of hearing damage in concert venues. Bull World Health Organ. 2020 May 1;98(5):367–369 

On page 368 and 369, the citation date for all references should read as follows: [cited 2020 Jan 18].

